# Association of PD-1 and PD-L1 Genetic Polymorphyisms with Type 1 Diabetes Susceptibility

**DOI:** 10.1155/2018/1614683

**Published:** 2018-11-11

**Authors:** Chenyue Qian, Heming Guo, Xiaohong Chen, Aiming Shi, Sicheng Li, Xin Wang, Jie Pan, Chen Fang

**Affiliations:** ^1^Department of Pharmacy, The Second Affiliated Hospital of Soochow University, Suzhou 215004, China; ^2^Department of Endocrinology, The Second Affiliated Hospital of Soochow University, Suzhou 215004, China; ^3^Department of Endocrinology, Jiangsu Province Hospital of TCM, 155 Hanzhonglu, Jiangsu Nanjing 210029, China

## Abstract

**Aims:**

The programmed death- (PD-) 1/PD-1 ligand (PD-L) pathway plays an important role in regulating T cell activation and maintaining peripheral tolerance. Accumulated studies showed that PD-1/PD-L1 pathway was involved in the development of type 1 diabetes (T1DM). Since the genetic background of type 1 diabetes differs greatly among the different population, we aim to investigate the association of genetic polymorphisms in PD-1 and PD-L1 with T1DM susceptibility in Chinese population.

**Methods:**

In total, 166 T1DM patients and 100 healthy controls were enrolled into the study. Genomic DNA was extracted from 4 mL peripheral blood samples collected from each subject. Genotyping of 8 selected SNPs of PD-1 and PD-L1 was carried out by the pyrosequencing PSQ 24 System using PyroMark Gold reagents (QIAGEN).

**Results:**

SNP rs4143815 in PD-L1 was significantly associated with T1DM. People carrying the C allele of rs4143815 suffering less risk of T1DM and T1DM patients with G/G genotype showed higher levels of autoantibody (AAB) positive incidence compared with C allele carriers. No significant associations were found in other SNPs.

**Conclusions:**

Our results indicate that rs4143815 of PD-L1 is significantly associated with T1DM and may serve as a new biomarker to predict the T1DM susceptibility.

## 1. Introduction

The increased prevalence of diabetes mellitus is considered one of the greatest public health challenges nowadays. Type 1 diabetes mellitus (T1DM), a polygenic autoimmune disease, is resulted from both genetic and environmental factors [1]. Although T1DM has a lower prevalence compared with type 2 diabetes mellitus (T2DM), it is the most common form of diabetes in childhood and has a greater impact on the quality of living life.

Programmed cell death 1 (PD-1) is an immunoinhibitory factor belonging to the CD28/B7 family. It plays a vital role in regulating T cell activation and maintaining peripheral tolerance as a core costimulatory molecule [2, 3]. Recently, PD-1 has been widely studied as an immune checkpoint that is applied to the treatment of numerous advanced cancers [4–6]. Programmed death ligand-1 (PD-L1) has been shown to be overexpressed in many cancers, including gastric cancer [7], esophageal cancer, pancreatic cancer, and other human gastrointestinal tumors [8]. Accumulated studies showed that blockage of the interaction between PD-1 and PD-L1 can help with better prognosis in various malignant tumors [6, 9, 10]. However, autoimmune diabetes has been reported after receiving anti-PD-1 therapy for tumor in both mouse models and human cases [11–13]. Thus, we assume that there may be a connection between PD-1/PD-L1 pathway and autoimmune diabetes.

Increasing studies had been committed to the association with PD-1/PD-L1 and autoimmune disease, including systemic lupus erythematosus, ankylosing spondylitis, allergic bronchial asthma, and autoimmune diabetes [14–17]. The role of PD-1 in T1DM has been studied using animal models. As a costimulatory molecule that inhibits T cell proliferation, PD-1 deficiency was shown to increase the risk of T1DM in nonobese diabetic (NOD) mice [18]. Studies had shown that low PD-1 might increase T cell proliferation and activation which lead to the destruction of beta cells, providing a possible mechanism for T1DM. Lower PD-1 expression was proposed to have associations with the development of T1DM in mouse models [19]. PD-L1 recently had been found expressed in the islets of people with type 1 diabetes [20], and we also found that PD-L1 was significantly reduced in the serum of T1DM patients [21]. Since single nucleotide polymorphisms (SNPs) play vital roles in the transcription and translation of genes and have associations with the occurrence and development of diseases, studies had been devoted to the associations between gene polymorphisms with T1DM susceptibility. Existing researches pointed out that PD-1 and PD-L1 SNPs were associated with T1DM susceptibility in different populations [21–23].

However, there is only a few studies focus on the associations between PD-1/PD-L1 gene polymorphisms and T1DM in Chinese population. Therefore, in the present study we investigated 8 selected SNPs of PD-1 and PD-L1 to ascertain whether PD-1 and PD-L1 SNPs influence the T1DM susceptibility.

## 2. Materials and Methods

### 2.1. Study Population

A total of 166 T1DM patients and 100 healthy controls were recruited for the study (Table 1). Blood samples were collected after overnight fasting from patients at the Endocrinology Department in the Second Affiliated Hospital of Soochow University, Suzhou, China, between 2013 and 2017. All the T1DM patients were diagnosed following the criteria of the American Diabetes Association (reference from Diabetes Care published by ADA). Samples from healthy blood donors, self-report healthy, were chosen as matched controls. Prior to commencing this study, the approval from the Ethics Review Board of the Second Affiliated Hospital of Soochow University was granted.

### 2.2. Selection of SNPs and Genotyping

Eight SNPs in 2 genes (PD-1 and PD-L1) were selected from the National Center for Biotechnology Information (NCBI) database (Table 2). All of the SNPs satisfied the following criteria: (1) SNPs that are associated with autoimmune disease occurrence or development according to the results of existing research; (2) SNPs that may influence function or expression of PD-1 and PD-L1 by NCBI; (3) minor allele frequency (MAF) ≥ 5% in Chinese Han population.

4 mL peripheral blood samples was collected in EDTA anticoagulant tubes for each patient. Genomic DNA was extracted using Genomic DNA Purification Kit according to the standard protocols. Genotyping of each polymorphism in the PD-1 and PD-L1 genes was carried out by the pyrosequencing PSQ 24 System using PyroMark Gold reagents (QIAGEN). Primers were designed using Pyrosequencing™ Assay Design Software and Primer Premier 5. The genomic region of interest was amplified by polymerase chain reaction (PCR) using one regular primer and one biotinylated primer; PCR products were sequenced using sequencing primer on PSQ 24 System. All the primers used in the present study were shown in Table 3.

### 2.3. Statistical Analysis

The chi-squared test was used to evaluate the Hardy-Weinberg equilibrium for allele frequencies of all SNPs. Unconditional logistic regression was conducted to calculate the adjusted odds ratio (OR) and 95% confidence intervals (95% CI). The associations between genotypes and T1DM susceptibility were tested using logistic regression analysis with Plink under additive, dominant, and recessive models. The test of significance was set at two-tailed *P* = 0.05.

The secondary structure of the mRNA was simulated using online webserver RNAfold (http://rna.tbi.univie.ac.at/cgi-bin/RNAWebSuite/RNAfold.cgi), which is a recognized software providing structure prediction service [24].

## 3. Results

166 T1DM patients and 100 healthy controls were listed into the present study. All subjects were Chinese and had a mean (SD) age of 34.1 ± 15.9 at the time of recruitment. 8 SNPs were genotyped in these subjects, only five of which conformed to Hardy-Weinberg equilibrium (*P* > 0.05) (shown in Table 2). Thus, we took rs2227981, rs34819629, rs4143815, rs2297136, and rs2297137 into final analyses. The results were summarized in Table 4.

There was a significant association between the PD-L1 SNP rs4143815 and T1DM susceptibility in the additive model (OR 0.50; *P* = 0.003), dominant model (OR 0.31; *P* = 0.012), and the recessive model (OR 0.46; *P* = 0.028). No significant association was observed between other SNPs and T1DM susceptibility. Considering multiple-testing correction, we calculated the *P* value for the SNPs by false detection rate (FDR) for correction. According to these results, rs4143815 remained significant in the additive model (*P* = 0.045).

In an exploratory analysis, clinical characteristics related to T1DM were compared among different genotypes of PD-L1 rs4143815 (shown in Table 5). Age, gender, HbA1c, LDL, and HDL did not vary considerably among genotypes of the rs4143815 SNP. However, the presence of subjects tested for autoantibodies (AABs) was higher in T1DM patients who were homozygous for the rs4143815G/G genotype compared with C allele carriers (*P* = 0.033).

**Figure 1:**
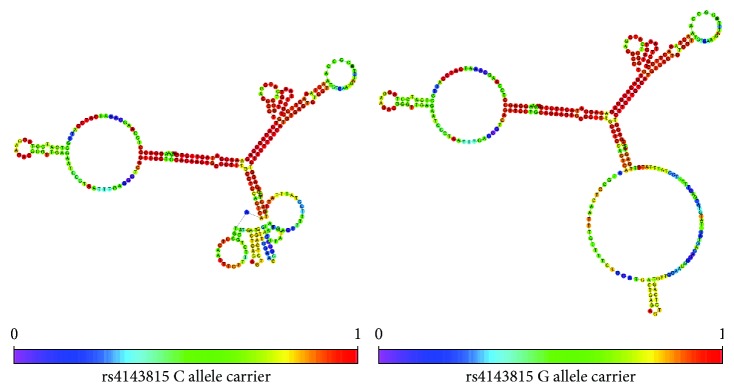
The secondary structure of partial PD-L1 3′UTR mRNA with different allele carrier of rs4143815 using RNAfold (http://rna.tbi.univie.ac.at/cgi-bin/RNAWebSuite/RNAfold.cgi).

We also simulated the secondary structure of partial PD-L1 3′UTR mRNA with different allele carrier using RNAfold (Figure 1). Different allele carrier of rs4143815 makes a great difference to the stem-loop structure of PD-L1 3′UTR, and this may account for its different binding force between miR-570 and mRNA leading to altered expression of PD-L1 protein and different susceptibility to T1DM.

## 4. Discussion

For the past few years, increasing studies had been committed to the associations with gene polymorphisms and T1DM susceptibility. The present study was made to investigate whether genetic polymorphisms in immune checkpoints could predict the onset of T1DM. In total, 266 subjects were included in the present study. With 8 selected SNPs genotyped, PD-L1 SNP rs4143815 remained significantly associated with T1DM susceptibility after FDR correction (additive model: *P* = 0.045). No significant associations were observed with other SNPs. Furthermore, T1DM patients homozygous for the rs4143815 G/G genotype showed higher levels of AAB positive incidence compared with C allele carriers (*P* = 0.033).

PD-1 is a core costimulatory molecule expressed by T cells that interact with its ligands PD-L1 and PD-L2 on antigen-presenting cells. PD-L1 was shown to be overexpressed in human gastrointestinal tumors [8]. PD-L1 negatively regulated immune response by interacting with PD-1 on T cells and therefore promoting T cell apoptosis [25]. Anti-PD-1 antibody treatment has been applied to the treatment of numerous advanced cancers; however, insulin-dependent diabetes had been reported during the anti-PD-1 immunotherapy process in both mouse models and human cases [11–13].

Increasing studies had been committed to the association with PD-1/PD-L1 and type 1 diabetes; low serum level of PD-1 and PD-L1 was reported being associated with T1DM in Japanese and Chilean patients [3, 21]. Association of polymorphisms of the PD-1and PD-L1 with T1DM has been evaluated previously in other countries [21–23]. Nevertheless, the genetic background of type 1 diabetes differs greatly among the different population.

In the present study, rs4143815 was found being associated with T1DM susceptibility. People carrying the C allele of rs4143815 suffering less risk of T1DM and T1DM patients with G/G genotype showed higher levels of AAB positive incidence compared with C allele carriers. Since the level of AABs has high associations of T1DM, the results of AABs reinforce the importance of rs4143815. People carrying the C allele of rs4143815 suffer lower AAB level and less risk of T1DM. However, HbA1c did not vary considerably among genotypes of the rs4143815 SNP. Since HbA1c is an important indicator for glycemic control and has little relevance to the susceptibility of the disease, this may be induced by the different treatment stage of T1DM patients.

It had been reported that rs4143815 was associated with the increased risk of gastric cancer [26, 27] and non-small-cell lung cancer [28, 29], possibly resulted from the suppression of immunological tumor surveillance by increased PD-L1 expression [30]. rs4143815 was also found being associated with the risk of T1DM in Chile [21]. This previous study comprised 205 T1DM patients and 205 normal children; however, the HWE equilibrium of rs4143815 for T1DM was 0.006. Our results use pyrosequencing to genotype all the SNPs, and the HWE equilibrium of rs4143815 is 0.752. With proper HWE equilibrium and advanced genotyping method, our results reinforce the importance of rs4143815 in T1DM susceptibility in Chinese population.

The rs4143815 is located in the 3′UTR of PD-L1 and binds to miR-570 [26]. Different allele may change the secondary structure of PD-L1 3′UTR. RNAfold was used to predict the secondary structure of PD-L1 3′UTR; G allele carrier shows a bigger stem-loop structure compared with C allele (shown in Figure 1) which may increase the interaction between PD-L1 3′UTR and miR-570 leading to lower PD-L1 expression. Lee et al. found that rs4143815 G allele exhibited a decreased transcription activity compared with C allele though luciferase report [31] which accords with our previous prediction.

Several studies had been committed to the association with PD-1 and autoimmune disease. PD 1.2, PD1.3, PD1.5, and PD1.9 were found being associated with systemic lupus erythematosus, ankylosing spondylitis, and allergic bronchial asthma [14–17]. However, our results showed that there are no associations between PD-1 polymorphisms and T1DM susceptibility. This may be due to different pathogenesis between T1DM and other autoimmune diseases.

Our study has some limitations. With limited SNPs and small sample size, the final result may have a certain deviation. We will select more typical SNPs and increase the sample size in the next step, and we will use gene chip and conduct multicenter studies to revalidate our results in the future.

## 5. Conclusion

In this study, our results indicate that rs4143815 of PD-L1 is significantly associated with T1DM susceptibility. Importantly, this SNP remained significantly after FDR correction. Furthermore, the presence of AABs is higher in T1DM patients homozygous for the rs4143815 G/G genotype compared with C allele carriers.

## Figures and Tables

**Table 1:** Characteristics of T1DM patients and normal controls in this study.

Characteristic	All	Type 1 diabetes	Control
Total number	266	166	100
Gender			
Female (*n*)	146	82	64
Male (*n*)	120	84	36
Age (year)	34.1 ± 15.9	25.9 ± 12.3	47.7 ± 11.1
Diabetes duration (year)	—	7.3 ± 6.5	—
HbA1c (%)	—	8.7 ± 2.8	—
Presence of AABs (IAA, ICA, and GADA)	—	97	—

Data are mean—standard deviation (SD); HbA1c: glycated hemoglobin; AABs: subjects tested for autoantibodies; IAA: insulin autoantibody; ICA: islet cell autoantibody; GADA: glutamic acid decarboxylase autoantibody.

**Table 2:** Eight single nucleotide polymorphisms examined in the present study.

Gene	SNP	Chromosome	Position	Mutation	MAF	HWE
PDCD1	rs34819629	2:241852468	Intron	G/A	A = 0.44	*P* = 0.170
	rs11568821	2:241851760	Intron	C/T	C = 1.0	*P* < 0.001
	rs10204525	2:241850169	Intron	C/T	C = 0.50	*P* = 0.002
	rs2227982	2:241851281	Intron	G/A	A = 0.60	*P* < 0.001
	rs2227981	2:241851121	Intron	G/T	T = 0.25	*P* = 0.758

PD-L1	rs2297136	9:5467955	3′UTR	A/G	G = 0.16	*P* = 0.197
	rs4143815	9:5468257	3′UTR	C/G	C = 0.46	*P* = 0.752
	rs2297137	9:5465732	Intron	G/A	A = 0.49	*P* = 0.845

SNP: single nucleotide polymorphism; MAF: minor allele frequency; HWE: Hardy-Weinberg equilibrium.

**Table 3:** All the primers used in the present study.

rs Number	Primer	Sequence
rs34819629	F	GTCCTGCACCTGGGGAATG
	R-Biotin	TCTGGAAGGGCACAAAGGTC
	Sequencing primer	ACCTGGGGAATGGTG

rs11568821	F	CCCCAGGCAGAACCTCAAT
	R-Biotin	GACCGCAGGCAGGCACATAT
	Sequencing primer	CCCCAGCCCACCTGC

rs10204525	F-Biotin	CTGACTCCCTCTCCCTTTCTC
	R	AAATCCAGCTCCCCATAGTCC
	Sequencing primer	GAGAACACAGGCACG

rs2297137	F	GCAAAGGCATTCCACTGTTC
	R-Biotin	ACCCCTTACGCTTCATCTTCAC
	Sequencing primer	GCATTCCACTGTTCAA

rs2227981	F	TTTCCAGTGGCGAGAGAAGA
	R-Biotin	GGCCAAGAGCAGTGTCCA
	Sequencing primer	CCGCCCGCAGGGGCT

rs2297136	F	ACGTAATCCAGCATTGGAACTT
	R-Biotin	TTCAGTGCTTGGGCCTTTTAA
	Sequencing primer	CAAGAGGAAGGAATGG

rs4143815	F	CTTTGCCTCCACTCAATGC
	R-Biotin	TACTGTCCCGTTCCAACACTG
	Sequencing primer	ACTCAATGCCTCAATTT

rs2227982	F	GGTTCGGTGCCGGTACTG
	R-Biotin	GGTCTTCTCTCGCCACTGGA
	Sequencing primer	CAAAGAAGGAGGACCC

**Table 4:** Association of five single nucleotide polymorphisms with type 1 diabetes susceptibility in all subjects.

Gene	SNP	Model	OR (95% CI)	*P* value
PD-1	rs2227981	ADD	1.05 (0.64 ~ 1.72)	0.846
		DOM	0.98 (0.52 ~ 1.84)	0.941
		REC	1.45 (0.41 ~ 5.12)	0.563
	rs34819629	ADD	0.74 (0.48 ~ 1.13)	0.163
		DOM	0.62 (0.30 ~ 1.27)	0.190
		REC	0.70 (0.34 ~ 1.43)	0.326

PD-L1	rs4143815	ADD	0.50 (0.31 ~ 0.79)	0.003^∗^
		DOM	0.31 (0.13 ~ 0.77)	0.012^∗^
		REC	0.46 (0.23 ~ 0.92)	0.028^∗^
	rs2297136	ADD	0.83 (0.48 ~ 1.43)	0.504
		DOM	0.76 (0.39 ~ 1.48)	0.421
		REC	1.00 (0.21 ~ 4.76)	0.995
	rs2297137	ADD	0.82 (0.53 ~ 1.28)	0.388
		DOM	0.61 (0.29 ~ 1.30)	0.203
		REC	0.97 (0.46 ~ 2.05)	0.930

ADD: additive model; DOM: dominant model; REC: recessive model; additive model: comparing the minor allele with major allele subjects; dominant model: comparing carriers of the minor allele with the major homozygous subjects; recessive model: comparing carriers of the major allele with the minor homozygous subjects.

**Table 5:** Clinical characteristics of T1DM patients in different genotype of rs4143815.

	PD-L1 rs4143815			
	G/G (*n* = 43)	G/C (*n* = 85)	C/C (*n* = 34)	*P* value
Age (years)	25.7 ± 11.6	26.8 ± 13.5	24.5 ± 10.5	0.661
Gender (% males)	46.5	55.3	44.1	0.447
HbA1c (%)	9.0 ± 2.5	8.6 ± 2.8	8.6 ± 2.7	0.737
LDL	2.7 ± 1.2	2.6 ± 0.7	2.6 ± 0.6	0.565
HDL	1.5 ± 0.5	1.6 ± 0.6	1.6 ± 0.4	0.212
Presence of AABs (IAA, ICA, GADA) (%)	72.1	49.4	67.6	0.033^∗^

Data are mean—standard deviation (SD); HbA1c: glycated hemoglobin; LDL: low-density lipoprotein; HDL: high-density lipoprotein; AABs: subjects tested for autoantibodies; IAA: insulin autoantibody; ICA: islet cell autoantibody; GADA: glutamic acid decarboxylase autoantibody.

## Data Availability

The data used to support the findings of this study are available from the corresponding author upon request.
